# Low psoas muscle index is a poor prognostic factor for lower gastrointestinal perforation: a single-center retrospective cohort study

**DOI:** 10.1186/s12893-019-0629-y

**Published:** 2019-11-28

**Authors:** Hajime Kayano, Eiji Nomura, Rin Abe, Yasuhiko Ueda, Takashi Machida, Chikara Fujita, Shohei Uchiyama, Kazuyuki Endo, Katsuki Murakami, Masaya Mukai, Hiroyasu Makuuchi

**Affiliations:** 1 Departments of General and Gastroenterological Surgery, Tokai University Hachioji Hospital, 1838 Ishikawa-machi, Hachioji, Tokyo, 192-0032 Japan; 2 Departments of Radiation Technology, Tokai University Hachioji Hospital, 1838 Ishikawa-machi, Hachioji, Tokyo, 192-0032 Japan

**Keywords:** Lower gastrointestinal perforation, Body composition, Psoas muscle index, Poor prognosis

## Abstract

**Background:**

Various body composition indices have been reported as prognostic factors for different cancers. However, whether body composition affects prognosis after lower gastrointestinal tract perforation requiring emergency surgery and multidisciplinary treatment has not been clarified. This study examined whether body composition evaluations that can be measured easily and quickly from computed tomography (CT) are useful for predicting prognosis.

**Methods:**

Subjects comprised 64 patients diagnosed with perforation at final diagnosis after emergency surgery for a preoperative diagnosis of lower gastrointestinal tract perforation and penetration. They were divided into a survival group and a non-survival (in-hospital mortality) group and compared. Body composition indices (psoas muscle index (PMI); psoas muscle attenuation (PMA); subcutaneous adipose tissue index (SATI); visceral adipose tissue index (VATI); visceral-to-subcutaneous fat area ratio (VSR)) were measured from preoperative CT. Cross-sectional psoas muscle area at the level of the 3rd lumbar vertebra was quantified. Optimal cut-off values were calculated using receiver operating characteristic curve analysis. Poor prognostic factors were investigated from multivariate logistic regression analyses that included patient factors, perioperative factors, intraoperative factors, and body composition indices as explanatory variables.

**Results:**

The cause of perforation was malignant disease in 12 cases (18.7%), and benign disease in 52 cases (81.2%). The most common cause was diverticulum of the large intestine. Emergency surgery for the 64 patients led to survival in 52 patients and death in 12 patients. On multivariate logistic regression analysis, independent predictors of poor prognosis were Sequential Organ Failure Assessment score (odds ratio 1.908; 95% confidence interval (CI) 1.235–3.681; *P* = 0.0020) and PMI (odds ratio 13.478; 95%CI 1.342–332.690; *P* = 0.0252). The cut-off PMI was 4.75 cm^2^/m^2^ for males and 2.89 cm^2^/m^2^ for females. Among survivors, duration of hospitalization was significantly longer in the low PMI group (29 days) than in the high PMI group (22 days, *p* = 0.0257).

**Conclusions:**

PMI is easily determined from CT and allows rapid evaluation of prognosis following lower gastrointestinal perforation.

## Background

Perforation of the lower gastrointestinal tract is a pathological condition that can easily result in severe bacterial infection and subsequent septic shock due to fecal peritonitis. As this condition increases in severity, disseminated intravascular coagulation (DIC) can arise and easily fall into multiple organ failure, sometimes leading to death. The basic principle of treatment is abdominal lavage and drainage with emergency surgery, focal excision, or colostomy. Furthermore, intensive treatment including blood purification therapies such as polymyxin-B direct hemoperfusion (PMX-DHP) and continuous hemodiafiltration (CHDF) is required after surgery, but the mortality rate remains high despite progress in surgical techniques and postoperative management [1, 2]. The ability to predict the prognosis before surgery would allow for more thorough and proactive perioperative management. Prediction of postoperative complications using various evaluations of body composition has recently been reported in gastrointestinal cancer surgery [3–5]. Further, sarcopenia has been reported as an important prognostic factor after abdominal surgery, including liver transplantation [6, 7]. However, with lower gastrointestinal tract perforation, very few studies have examined the influence of body components such as skeletal muscle mass or fat mass on prognosis. The purpose of this study was therefore to clarify whether somatic composition affects the prognosis of lower gastrointestinal perforation.

## Methods

### Study design

This retrospective cohort study was conducted at Tokai University Hachioji Hospital after being approved by the Institutional Review Board for Clinical Research at Tokai University. A total of 83 patients were diagnosed with perforation and penetration of the lower gastrointestinal tract from May 2010 to March 2019 and were treated with emergency surgery. Two cases for which abdominal computed tomography (CT) was not performed before surgery were excluded from this study. A further 17 cases in which the patient was diagnosed with lower digestive tract penetration from both preoperative abdominal CT and intraoperative findings were excluded from this study because they did not have panperitonitis and may not always be indicated for surgery. The remaining 64 cases were divided into a survival group and a non-survival (in-hospital mortality) group and analyzed.

### Data collection

Patient factors, preoperative factors, intraoperative factors, and postoperative courses are managed in a database. CT examinations performed preoperatively are stored in electronic medical records. Data from these sources were accessed for this study.

### Patient and perioperative factors

Patient factors included age, sex, preoperative comorbidities, and body mass index (BMI). Preoperative factors included white blood cell count, C-reactive protein (CRP) concentration, albumin value, time from onset to surgery, Acute Physiology and Chronic Health Evaluation (APACHE) II score [8], and Sequential Organ Failure Assessment (SOFA) score [9]. Intraoperative factors included operative time, blood loss, presence or absence of blood transfusion, cause of perforation, site of perforation, and operation method.

### Body component analysis measurement

**Fig. 1:**
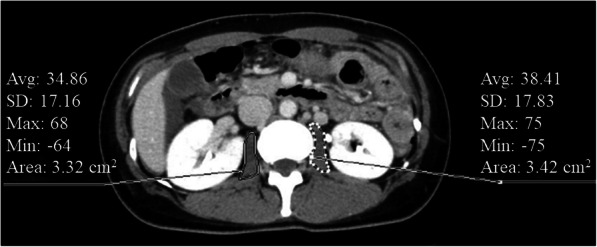
PMI measurement. Body muscle mass measured using axial CT imaging at the level of the third lumbar vertebra (L3)

Abdominal CT images used in the diagnosis of lower gastrointestinal perforation before surgery were used to evaluate body composition. Abdominal CT was performed using an Aquilion ONE™ platform (Canon Medical Systems, Tochigi, Japan). Psoas muscle mass, CT attenuation value of the psoas muscle, visceral fat area, and subcutaneous fat area were analyzed using a Ziostation2 Plus general-purpose diagnostic imaging workstation (Ziosoft, Tokyo, Japan). Each component was measured from horizontal cross-sections of the abdominal CT images. Psoas muscle area was traced as the region of interest (ROI) of the iliopsoas muscle contour at the level of the third lumbar vertebra (L3) (Fig. 1), and the sum of left and right areas was calculated. This area was then standardized as the psoas muscle index (PMI; in cm^2^/m^2^) by dividing the value by the square of height in meters. In addition, the CT attenuation value of the psoas muscle traced as described above was obtained to represent the variable of fat accumulation in psoas muscle that characterizes muscle atrophy, and the average of values for left and right muscles was taken as psoas muscle attenuation (PMA; in Hounsfield units). In the same manner, the areas of subcutaneous fat and visceral fat were calculated by measuring abdominal fat mass using the horizontal cross-section at the L3 level, and each area was then divided by the square of height in meters. With these standardizations, subcutaneous adipose tissue index (SATI; in cm^2^/m^2^) and visceral adipose tissue index (VATI; in cm^2^/m^2^) were determined, respectively. In addition, visceral-to-subcutaneous fat area ratio (VSR) was calculated as the visceral fat area divided by the subcutaneous fat area as an index of abdominal fat distribution. The receiver operating characteristic (ROC) curve was plotted for each variable, the optimal cut-off value for death due to lower gastrointestinal perforation was obtained via the Youden Index, and participants were assigned to the low-value or high-value group accordingly.

### Postoperative multidisciplinary treatment

Postoperative multidisciplinary treatment was evaluated by the presence or absence of ventilatory management, blood purification (PMX-DHP), recombinant human soluble thrombomodulin (rTM) (Asahi Kasei Pharma Corporation, Tokyo, Japan), human anti-thrombin III, freeze-dried concentrated (hATIII) (Japan Blood Products Organization, Tokyo, Japan), and intravenous immunoglobulin (IVIg) (freeze-dried sulfonated human normal immunoglobulin; Teijin Pharma, Tokyo, Japan).

### Outcome data

Data reflecting the postoperative course included DIC score on postoperative day 1, presence or absence of DIC on postoperative day 1, length of stay (LOS) in the intensive care unit (ICU) in days, duration until withdrawal of ventilatory support in days, postoperative complications, and postoperative stay in days. All postoperative complications were classified using the Clavien-Dindo classification (CD) [10], with CD3 or above defined as postoperative complications for the purposes of this study.

### Statistical analysis

In the statistical analyses, comparisons between two groups were made using the Mann-Whitney test for continuous variables and either a χ^2^ test or Fisher’s exact test (for any test involving a low number of samples) for categorical variables, with values of *p* < 0.05 regarded as significant. The determination of each cut-off was determined from the maximal Youden index for the ROC curve. Separate determinations were made for males and females, as muscle mass is significantly greater in males than in females regardless of age [11]. To identify prognostic factors, univariate analysis was performed first, followed by multivariate analysis using all significant variables from univariate analysis. On univariate analysis, the Mann-Whitney test was used for continuous variables, and the Pearson χ^2^ test for categorical variables. Multivariate analysis was performed using logistic regression. The software used for all statistical analyses was JMP for Windows version 13.0 (SAS Institute, Cary, NC).

## Results

### Patient characteristics

The 64 patients included more females than males, with a median age of 72 years (Table 1). Preoperative comorbidities were noted in 47 cases (73.4%), with hypertension as the most common, followed by heart disease and cerebrovascular disease. Median APACHE II score and SOFA score on admission to the ICU were 11 and 4, respectively. Median time from onset to surgery was 10 h, median operative time was 148 min, and median blood loss was 244 ml, with blood transfusion performed in 37.5% of cases. In terms of body composition, PMI, VAR, and PMA were higher in males than in females, but SATI and VATI were higher in females than in males (Table 1). The cause of perforation was malignant disease in 12 cases (18.7%) and benign disease in 52 cases (81.2%). The most common cause of perforation was diverticulum of the large intestine, followed by iatrogenic perforation. The site of perforation involved the right colon in 10 cases and the left colon in 54 cases, with the sigmoid colon accounting for 36 cases (56.2%). More than half of the cases (51.6%) underwent the Hartmann operation, followed by colon colostomy only.

**Table 1:** Baseline characteristics

Characteristic	*n = 64*
Patient characteristics	
Age (years)^a^	72 (46–101)
Sex, male/female, n (%)	25 (39.0) / 39 (60.9)
BMI (kg/m^2^)^a^	21.2 (13.5–36.0)
Comorbidities, n (%)	47 (73.4)
Hypertension	33 (51.5)
Heart disease	24 (37.5)
Diabetes mellitus	10 (15.6)
Cerebrovascular disease	10 (15.6)
Renal disease	9 (14.0)
Lung disease	6 (9.3)
Collagen disease	5 (7.8)
Mental disease	3 (4.6)
Perioperative characteristics	
White blood cells (/μl)^a^	8000 (900–509,000)
C-reactive protein (mg/dl)^a^	4.8 (0.01–40.9)
Platelet (×10^4^/μl)^a^	21.7 (3.9–75.4)
Albumin (mg/dl)^a^	2.9 (1.6–4.7)
APACHE II score^a^	11 (2–22)
SOFA score^a^	4 (0–14)
Interval from onset to surgery (h)^a^	10 (2–200)
Intraoperative factor	
Primary disease, n (%)	
Diverticulosis	26 (40.6)
Iatrogenic	13 (20.3)
Cancer	12 (18.7)
Fecal impaction	8 (12.5)
Ischemic	5 (7.8)
Site of perforation, n (%)	
Sigmoid	36 (56.2)
Rectum	10 (15.6)
Descending	8 (12.5)
Ascending	6 (9.3)
Transverse	2 (3.1)
Cecum	2 (3.1)
Surgical procedure, n (%)	
Hartmann operation	33 (51.5)
Colostomy	10 (15.6)
Resection with ileostomy	6 (9.3)
Resection with colostomy	5 (7.8)
Simple closure with colostomy	5 (7.8)
Resection	3 (4.6)
Simple closure with ileostomy	2 (3.1)
Operative time (min)^a^	148 (30–336)
Blood loss (ml)^a^	244 (5–4100)
Transfusion (%), n (%)	24 (37.5)
Body composition variables	
PMI (cm^2^/m^2^)	3.60 (1.42–7.02)
Male/Female	4.44 (1.59–6.81) / 3.09 (1.42–7.02)
SATI (cm^2^/m^2^)	32.72 (0.01–136.09)
Male/Female	22.48 (0.34–105.37) / 47.27 (0.01–136.09)
VATI (cm^2^/m^2^)	22.64 (0.53–112.77)
Male/Female	21.95 (1.83–98.06) / 23.33 (0.53–112.77)
VSR	0.73 (0.14–5.04)
Male/Female	0.75 (0.32–7.31) / 0.59 (0.14–5.04)
PMA (HU)	37.78 (−22.92–66.76)
Male/Female	44.30 (18.90–66.76) / 34.41 (−22.92–58.52)

*BMI* body mass index (kg/m^2^), *APACHE II* Acute Physiology and Chronic Health Evaluation II, *SOFA* Sequential Organ Failure Assessment, *PMI* psoas muscle index (cm^2^/m^2^), *SATI* subcutaneous adipose tissue index (cm^2^/m^2^), *VATI* visceral adipose tissue index (cm^2^/m^2^), *VSR* visceral-to-subcutaneous fat area ratio, *PMA* psoas muscle attenuation (HU), *HU* Hounsfield units

^a^Values are shown as median (range)

### Cut-off values and AUCs for body composition

Cut-off values and AUCs for body composition are shown in Table 2. Males showed higher cut-off values than females for all variables. The PMI for males was the highest AUC among males at 0.72. The PMI for females was 0.63, the second-highest AUC among females.

**Table 2:** Cut-off values and AUC for body composition

		PMI (cm^2^/m^2^)	SATI (cm^2^/m^2^)	VATI (cm^2^/m^2^)	VAR	PMA (HU)
Men	Cut-off	4.75	25.98	36.95	3.05	37.57
	AUC	0.72	0.56	0.57	0.59	0.67
Female	Cut-off	2.89	0.27	8.79	1.24	32.86
	AUC	0.63	0.62	0.57	0.68	0.56

*AUC* area under the curve

### Prognostic factors

In examining for prognostic factors (Table 3), no significant differences between the survival group and non-survival group were seen in patient factors. Among preoperative factors, platelet count (15.35 × 10^4^/μl vs. 22.45 × 10^4^/μl; *P* = 0.0040) and albumin levels (2.4 mg/dl vs. 3.0 mg/dl; *P* = 0.0082) were significantly lower in the non-survival group compared to the survival group. In addition, the non-survival group showed significantly higher APACHE II score (17 vs 10; *P* = 0.0007) and SOFA score (9 vs 3; *P* = 0.0001) than the survival group. No significant differences between groups were seen among intraoperative factors. Among body composition indices, the non-survival group showed significantly higher frequencies of low PMI (75% vs 40.38%; *P* = 0.0303) and low VAR (86.54% vs 58.33%; *P* = 0.0240) compared to the survival group. Using each factor that showed significant differences from univariate analyses, multivariate logistic regression analysis identified SOFA score (odds ratio 1.908, 95% confidence interval (CI) 1.235–3.681, *P* = 0.0020) and PMI (odds ratio 13.478, 95%CI 1.342–332.690, *P* = 0.0252) as independently associated with poor prognosis.

**Table 3:** Uni- and multivariate analysis of factors predicting using logistic regression

	Univariate analysis			Multivariate analysis		
	Survival (*n* = 52)	Non-survival (*n* = 12)	*P*-value	Odds ratio	95% CI	*P*-value
Patient factors						
Age (years)^a^	72 (46–101)	74 (60–87)	0.8029			
Sex, male/female, n (%)	18 (34.6) / 34 (65.3)	7 (58.3) / 5 (41.6)	0.1290			
BMI (kg/m^2^)^a^	21.6 (13.5–36.0)	20.4 (15.2–25.6)	0.2563			
Comorbidities, n (%)	36 (69.2)	11 (91.6)	0.1127			
Hypertension	25 (48.0)	8 (66.6)				
Heart disease	19 (36.5)	5 (41.6)				
Diabetes mellitus	8 (15.3)	2 (16.6)				
Cerebrovascular disease	6 (11.5)	4 (33.3)				
Collagen disease	5 (9.6)	0 (0)				
Renal disease	5 (9.6)	4 (33.3)				
Lung disease	4 (7.6)	2 (16.6)				
Mental disease	1 (1.9)	2 (16.6)				
Perioperative factors						
White blood cells (/μl)^a^	8550 (900–50,900)	3450 (1200–40,600)	0.3311			
C-reactive protein (mg/dl)^a^	4.6 (0.01–40.9)	13.3 (0.1–32.2)	0.3620			
Platelets (×10^4^/μl)^a^	22.4 (9.6–75.4)	15.3 (3.9–32.2)	0.0040^*^	0.898	0.771–1.012	0.0831
Albumin (mg/dl)^a^	3.0 (1.6–4.7)	2.4 (1.7–3.6)	0.0082^*^	0.495	0.179–33.052	0.5716
APACHE II score^a^	10 (2–22)	17 (5–21)	0.0007^*^	1.114	0.864–1.466	0.6986
SOFA score^a^	3 (0–10)	9 (5–14)	0.0001^*^	1.908	1.235–3.681	0.0020^*^
Interval from onset to surgery (h)^a^	10 (3–140)	8 (2–200)	0.1961			
Intraoperative factors						
Primary disease, n (%)			0.0764			
Diverticulosis	23 (44.2)	3 (25.0)				
Iatrogenic	11 (21.1)	2 (16.6)				
Cancer	11 (21.1)	1 (8.3)				
Fecal impaction	4 (7.6)	4 (33.3)				
Ischemic	3 (5.7)	2 (16.6)				
Site of perforation, n (%)			0.3245			
Sigmoid	32 (61.5)	4 (33.3)				
Rectum	8 (15.3)	2 (16.6)				
Descending	5 (9.6)	3 (25.0)				
Ascending	5 (9.6)	1 (8.3)				
Transverse	1 (1.9)	1 (8.3)				
Cecum	1 (1.9)	1 (8.3)				
Operative time (min)^a^	148 (30–287)	136 (82–336)	0.7660			
Blood loss (ml)^a^	215 (5–4100)	500 (5–3130)	0.3160			
Transfusion, n (%)	17 (32.6)	7 (58.3)	0.0982			
Surgical procedure, n (%)			0.4791			
Hartmann’s	27 (51.9)	6 (50.0)				
Colostomy	9 (17.3)	1 (8.3)				
Resection with colostomy	4 (7.6)	1 (8.3)				
Simple closure with colostomy	4 (7.6)	1 (8.3)				
Resection with ileostomy	3 (5.7)	3 (25.0)				
Resection	3 (5.77)	0 (0)				
Simple closure with ileostomy	2 (3.8)	0 (0)				
Body composition variable						
Low PMI, n (%)	21 (40.38)	9 (75.0)	0.0303^*^	13.478	1.342–332.690	0.0252^*^
Low VATI, n (%)	19 (36.54)	7 (58.33)	0.1658			
Low SATI, n (%)	10 (19.23)	5 (41.67)	0.0982			
Low VSR, n (%)	45 (86.54)	7 (58.33)	0.0240^*^	3.5075	0.190–83.333	0.3925
Low PMA, n (%)	15 (28.85)	5 (41.67)	0.3878			

^*^P < 0.05

*CI* confidence interval

^a^Values are shown as median (range)

### Influence of PMI on survival cases

In comparing the postoperative course and postoperative multidisciplinary treatment in the high and low PMI subgroups within the survival group, hospitalization was significantly longer in the low PMI subgroup (29 days) than in the high PMI subgroup (22 days, *p* = 0.0257) (Table 4). DIC score, DIC rate, postoperative complication rate, and LOS in the ICU all tended to be higher in the low PMI subgroup than in the high PMI subgroup. However, no difference in postoperative multidisciplinary treatment was found between the two subgroups (Table 5).

**Table 4:** Comparison of postoperative course according to PMI within the survival group

	Low PMI (*n* = 21)	High PMI (*n* = 31)	*P*-value
DIC score^a^	3 (0–6)	2 (0–6)	0.2149
DIC, n (%)	7 (33.33)	8 (25.81)	0.5566
Artificial ventilator, n (%)	8 (38.10)	15 (48.39)	0.4634
Ventilator weaning (days)^a^	3 (1–9)	3 (1–38)	0.4267
Complications 2/3, n (%)	7 (33.33)	7 (22.58)	0.3910
LOS in ICU (days)^a^	4 (1–19)	4 (1–18)	0.1791
LOS in hospital (days)^a^	29 (10–86)	22 (10–58)	0.0257^*^

^*^P < 0.05

DIC, disseminated intravascular coagulation; LOS, length of stay; ICU, intensive care unit

^a^Values are shown as median (range)

**Table 5:** Comparison of postoperative multidisciplinary treatment according to PMI within the survival group

	Low PMI (*n* = 21)	High PMI (*n* = 31)	*P*-value
Vasopressor, n (%)	7 (33.33)	10 (32.26)	0.9354
rTM, n (%)	10 (47.62)	13 (41.94)	0.6855
IVIg, n (%)	12 (57.14)	16 (51.61)	0.6947
hATIII, n (%)	3 (14.29)	5 (16.13)	0.8565
PMX, n (%)	7 (33.33)	8 (25.81)	0.5566

*rTM* recombinant human soluble thrombomodulin, *IVIg* intravenous human immunoglobulin, *hATIII* human anti-thrombin III, *PMX-DHP* polymyxin B-immobilized fiber column-direct hemoperfusion

## Discussion

The mortality rate for lower gastrointestinal perforation has been reported as 15.5–26.6% [1, 2, 12], similar to the rate in this study. This is probably largely attributable to the presence of numerous Gram-negative bacilli in the large intestine, so bacteremia easily arises following perforation of the lower gastrointestinal tract, and chemical transmitters such as interleukin (IL)-6 are induced, resulting in rapid onset of septic shock. This is considered to lead to multiple organ failure and acute circulatory failure. In abdominal emergencies requiring surgery, the frequency of lower gastrointestinal perforation is not particularly high [13, 14]. Identification of prognostic factors to improve survival rates for this pathology has long been a priority, due to the high mortality rate [15–17]. In addition, the severity of lower gastrointestinal perforation is considered to involve a large number of prognostic factors, and a scoring system is considered important for judging the preoperative condition more comprehensively. Methods for evaluating prognosis in patients with severe disease in general include APACHE II, the Simplified Acute Physiology Score III [18], and the Mortality Prediction Model 0 III [19] as overall evaluations of systemic severity, and multiple organ dysfunction score [20] and SOFA as an evaluation of multiple organ failure. The present study also showed significant differences in APACHE II from univariate analysis, and SOFA was an independent prognostic factor, confirming its usefulness as a severity assessment method. Moreover, Physiological and Operative Severity Score for Mortality and Morbidity (POSSUM) [21, 22] is available as a comprehensive evaluation of various organ functions and the degree of surgical invasion, indicating surgical risk. POSSUM is reported to be an excellent prognostic system even for colorectal peritonitis [23]. However, because these evaluation methods require a large number of items, they are overly complicated for cases of lower digestive tract perforation requiring emergency surgery and cannot be evaluated appropriately at all facilities. Identification of factors that can be evaluated quickly and easily is therefore necessary. In this study, low PMI and high SOFA score were independent prognostic factors in multivariate logistic regression analysis. PMI is a simple but reliable method of prognostic evaluation similar to SOFA. Body composition has recently been reported as a risk factor or prognostic factor for postoperative complications [3, 5, 24, 25]. In particular, skeletal muscle mass is considered important [26–28]. Among the methods of evaluating body composition that include skeletal muscle mass are bioelectrical impedance analysis (BIA), dual-energy X-ray absorptiometry (DXA), and CT/magnetic resonance imaging using cross-sectional images. BIA and DXA methods show a strong correlation with each other [29], as do the BIA and CT cross-sectional methods [30]. Each has its advantages and disadvantages. However, in the case of emergency diseases and gastrointestinal cancers, the CT method requires no additional examinations and is simple and quick if images from preoperative examinations are used. Furthermore, with CT methods, one method uses total skeletal muscle area at the L3 level and another measures only the psoas muscle area, but the results are reportedly correlated [30]. In the case of emergency diseases, measuring only psoas may be better in terms of simplicity and speed. The skeletal muscle system accounts for about 40% of the adult body volume, and around 88% of muscle is protein, which represents 50% of total protein in the body. The muscles function as a nutrient storage system, playing the role of distributing amino acids to each organ as a biological defense reaction during invasion. However, in patients with sepsis, active nutrition cannot prevent loss of the body protein compartment despite increases in body fat [31]. In addition, inflammatory cytokines such as IL-6 [32] and tumor necrosis factor α [33] promote proteolysis. Therefore, when extravasation results from emergency surgery under conditions of severe infection, a greater original muscle mass is advantageous for tissue repair as a defense reaction, and organ failure can be avoided. Preoperative skeletal muscle mass is a prognostic indicator in lower gastrointestinal perforation. On the other hand, many reports [27, 34] have identified the CT attenuation value as a useful index of muscle quality in cancer patients, because the increase in non-contractile tissue including fat in muscles decreases the CT attenuation value. In this study, CT attenuation was not a prognostic indicator. Under pathological conditions such as lower gastrointestinal perforation, where damage to the body is largely caused by the release of inflammatory cytokines, muscle mass is considered important regardless of muscle quality. In addition, none of visceral fat mass, subcutaneous fat mass or the ratio of those two values represented prognostic indicators. Muscle mass was considered more important in the acute phase than fat. Survivors with low PMI tended to have high DIC score, high DIC rate, and high postoperative complication rate, and additionally had significantly longer duration of hospitalization. It has been suggested that if skeletal muscle mass is reduced, later treatment will be difficult even if the patient survives. However, no significant difference in treatment methods was seen between the two survival groups.

Postoperative intensive care is important in lower gastrointestinal perforation. In intensive care, blood purification treatments such as CHDF and PMX-DHP are available, along with thrombomodulin alfa (a genetical recombination) as pharmacotherapy. No specific opinion has been obtained regarding prognostic improvements from these treatment modalities, and no clear criteria for the introduction of blood purification therapy have been established. Preoperative medical interventions for skeletal muscle loss are not possible with lower gastrointestinal perforation. To improve the survival rate, early introduction of aggressive blood purification and medication may be necessary in the muscle loss group. Measuring the psoas muscle area from CT may offer a useful method for patients who are not suffering from shock upon arrival at the hospital and in facilities where CT can be performed immediately anytime, 24 h a day. In this study, the number of cases was limited because of the single-center design, and the ability to conduct sufficient studies appears limited. To improve the number of cases in the future, multiple-center studies appear necessary. Furthermore, changes in the effects of treatment according to differences in skeletal muscle mass should be accurately examined in prospective studies.

## Conclusions

Decreased psoas muscle mass was independently associated with poor prognosis of lower gastrointestinal perforation. Measurement of psoas muscle area using CT is convenient, quick, and useful for estimating prognosis.

## Data Availability

The datasets used and/or analyzed during the current study are available from the corresponding author on reasonable request.

## Abbreviations

APACHE II: Acute Physiology And Chronic Health Evaluation II
AUC: Area under the curve
CHDF: Continuous hemodiafiltration
CT: Computed tomography
hATIII: Human anti-thrombin III
HU: Hounsfield units
IVIg: Intravenous human immunoglobulin
LOS: Length of stay
PMA: Psoas muscle attenuation
PMI: Psoas muscle index
PMX-DHP: Polymyxin B-immobilized fiber column-direct hemoperfusion
ROC: Receiver operating characteristic
rTM: Recombinant human soluble thrombomodulin
SATI: Subcutaneous adipose tissue index
SOFA score: Sequential Organ Failure Assessment
VATI: Visceral adipose tissue index
VSR: Visceral-to-subcutaneous fat area ratio
